# Supplementary figures and images for: Longitudinal trends in renal function among first time sugarcane harvesters in Guatemala
Source: PLoS One. 2020 Mar 6;15(3):e0229413. doi: 10.1371/journal.pone.0229413 (PMC7059928; doi:10.1371/journal.pone.0229413)

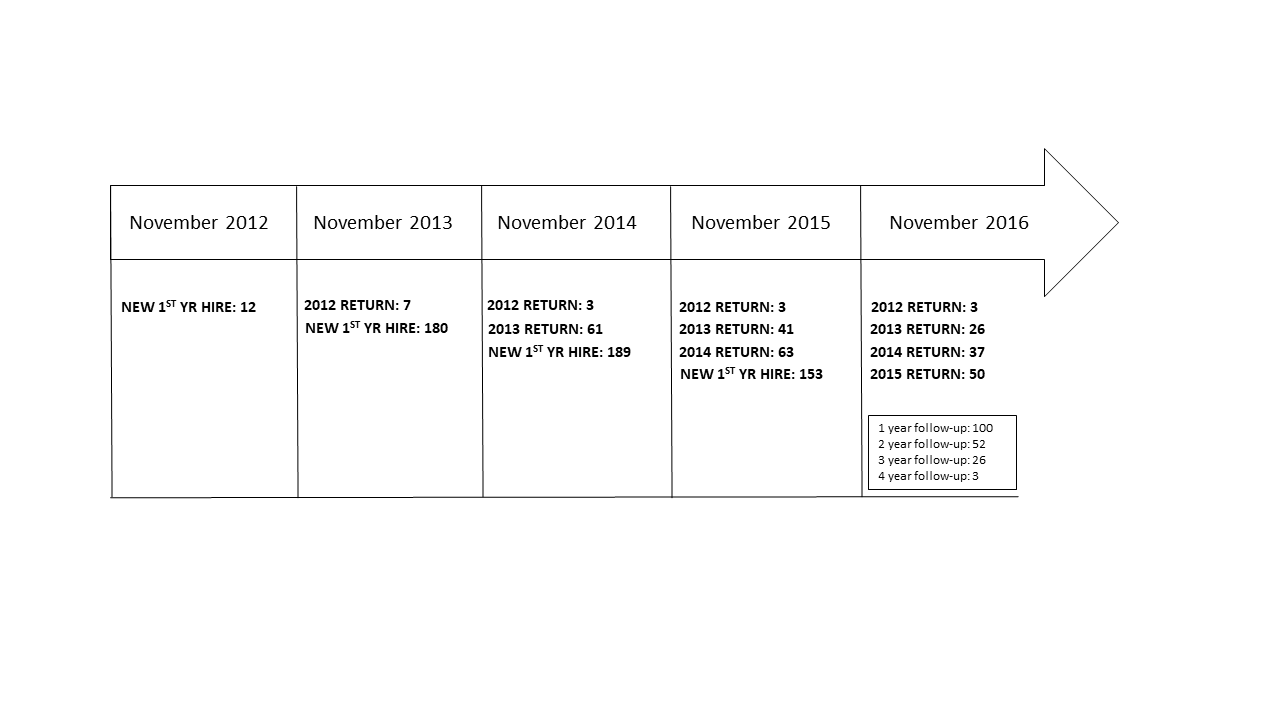

Supplement: S1 Fig — Study flow showing timeline along with the number of new workers screened and the number returning each subsequent year. (TIF) [file pone.0229413.s001.tif]
