# Supplementary material for: Longitudinal trends in renal function among first time sugarcane harvesters in Guatemala
Source: PLoS One. 2020 Mar 6;15(3):e0229413. doi: 10.1371/journal.pone.0229413 (PMC7059928; doi:10.1371/journal.pone.0229413)
Supplement: S1 File — (DOCX) [file pone.0229413.s002.docx]

**Supporting Information**

**Modelling approach and model selection**

Joint latent class mixed models combine two sub-models. The first is a linear mixed model for the underlying latent longitudinal change in eGFR. The second is the proportional hazard model for time-to-attrition. The two models are linked through unobserved latent classes, which account for heterogeneity and represent sub-populations with different shapes of longitudinal change in eGFR and risk of attrition [1]. Time was treated as season (1-5 harvest seasons) in the linear mixed model. To determine the functional form of time, linear mixed models with linear, quadratic, and cubic terms for time were compared using -2logliklihood tests. The longitudinal change in eGFR was modelled with a quadratic time trend with random terms for intercept and 2-degree polynomial time. A forward modelling approach was used to determine the number of optimal latent classes, starting with the one class solution. We incrementally added extra classes until convergence failed. Models were run specifying Weibull, piecewise constant with four knots, and cubic M-splines with four knots as the baseline risk function. The automatic choice of initial values was used. To select the appropriate baseline risk function and optimal latent classes we compared the Bayesian Information Criterion (BIC) from the resulting models. To verify that global maximum rather than local maximum was found, grid searches using 1000 random vectors of initial values and a maximum of 15 iterations were used for the best performing baseline risk function along the range of latent classes. The resulting class models were compared using BIC and the model with the lowest BIC was selected. The R package “lcmm” was used [2]. Posterior classification probabilities ranged from 91% to 96%.

**Cohort comparison**

First year sugarcane harvesters who were excluded from the analysis cohort had worked on average 16 of the 26 weeks of their first harvest indicating that they did not fully complete their first harvest. At the time that they entered the workforce, they were slightly older (24 years compared to 22 years; p-value <0.01) than those who returned. There were no other notable differences in baseline eGFR nor baseline health, demographics, or behavioral factors between those who met inclusion criteria and those who did not (See S1 Table).

**References**

1. Marioni RE, Proust-Lima C, Amieva H, Brayne C, Matthews FE, Dartigues J-F, et al. Social activity, cognitive decline and dementia risk: a 20-year prospective cohort study. BMC Public Health. 2015;15:1089-. doi: 10.1186/s12889-015-2426-6. PubMed PMID: 26499254.

2. Proust-Lima C, Philipps V, Liquet B. Estimation of Extended Mixed Models Using Latent Classes and Latent Processes: The R Package lcmm. 2017. 2017;78(2):56. Epub 2017-06-01. doi: 10.18637/jss.v078.i02.
