# Supplementary material for: Longitudinal trends in renal function among first time sugarcane harvesters in Guatemala
Source: PLoS One. 2020 Mar 6;15(3):e0229413. doi: 10.1371/journal.pone.0229413 (PMC7059928; doi:10.1371/journal.pone.0229413)
Supplement: S2 File — (DOCX) [file pone.0229413.s003.docx]

**Registro de Chequeo Médico Pre-empleo**

1. Datos de identificación
   1. Ficha
   2. DPI
   3. Cedula
   4. Fecha de evaluación
   5. Nombre y Apellidos
   6. Departamento
   7. Municipio
   8. Sexo (M o F)
   9. Edad (años)
   10. Fecha de nacimiento (dd/mm/aaaa)
2. Signos vitales
   1. Peso
   2. Talla
   3. Presión arterial
3. Fuente de agua
   1. Pozo
   2. Potable
   3. Otra (especificar)
4. Historia laboral
   1. Puesto a despeñar (especificar)
   2. Trabajó en ingenios
      1. Si
         1. Numero de zafras en Ingenio Pantaleon (#)
         2. Numero de zafras en Ingenio Concepcion (#)
         3. Numero de zafras en otros Ingenios (#)
      2. No
5. Alcohol
   1. Si
   2. No
6. Fumado
   1. Si
      1. Duración en años
   2. No
   3. Fumaba antes
      1. Si
         1. Duración en años
      2. No
7. Historia medica
   1. Hipertensión
      1. Si
      2. No
   2. Diabetes
      1. Si
      2. No
   3. Insuficiencia renal crónica
      1. Si
      2. No
8. Examen físico
   1. Cardiaco
      1. Si
      2. No
   2. Locomotor
      1. Deformidad, limitación o incapacidad
         1. Si
            1. Especifique la ubicación anatómica, el tipo y gravedad de la limitación
         2. No
9. Examenes de laboratorio
   1. biometría hemática completa (BHC)
   2. creatinina
10. Examenes especiales
    1. Agudeza visual
    2. Examenes periodicos recomendados (especificar)
