# Supplementary material for: Longitudinal trends in renal function among first time sugarcane harvesters in Guatemala
Source: PLoS One. 2020 Mar 6;15(3):e0229413. doi: 10.1371/journal.pone.0229413 (PMC7059928; doi:10.1371/journal.pone.0229413)
Supplement: S3 File — (DOCX) [file pone.0229413.s004.docx]

**Pre-employment Clinical Evaluation Form**

1. Identifying information
   1. Employee ID
   2. DPI
   3. ID number
   4. Date of evaluation
   5. Name
   6. Department
   7. Municipality
   8. Gender (M or F)
   9. Age (years)
   10. Date of birth (dd/mm/yyyy)
2. Vital signs
   1. Weight
   2. Height
   3. Blood pressure
3. Water source
   1. Well
   2. Piped
   3. Other (specify)
4. Work history
   1. Position held (specify)
   2. Previous work in sugarcane
      1. Yes
         1. # of zafras at Ingenio Pantaleon (#)
         2. # of zafras at Ingenio Concepcion (#)
         3. # of zafras at other mills (#)
      2. No
5. Alcohol
   1. Yes
   2. No
6. Smoking
   1. Yes current
      1. Duration in years
   2. No
   3. Smoked previously
      1. Yes
         1. Duration in years
      2. No
7. Medical history (completed by physician)
   1. Hypertension
      1. Yes
      2. No
   2. Diabetes
      1. Yes
      2. No
   3. Chronic kidney disease
      1. Yes
      2. No
8. Physical exam (completed by physician)
   1. Cardiac
      1. Yes
      2. No
   2. Locomotor
      1. Deformity, limitation or disability
         1. Yes
            1. Specify the anatomical location, type and severity of limitation
         2. No
9. Laboratory results
   1. Complete blood count
   2. Creatinine
10. Special exams
    1. Visual acuity
    2. Recommended periodic exams (specify)
