# Supplementary material for: Longitudinal trends in renal function among first time sugarcane harvesters in Guatemala
Source: PLoS One. 2020 Mar 6;15(3):e0229413. doi: 10.1371/journal.pone.0229413 (PMC7059928; doi:10.1371/journal.pone.0229413)
Supplement: S1 Table — (DOCX) [file pone.0229413.s005.docx]

|  | Included in analysis  (N = 181) | Excluded from analysis  (N = 353) | p-value |
| --- | --- | --- | --- |
| Creatinine, mg/dL | 0.88 (0.12) | 0.89 (0.17) | 0.392 |
| eGFR, ml/min per 1.73 m^2^ | 119.43 (12.97) | 116.48 (16.43) | 0.036 |
| Age, years | 22 (5) | 24 (7) | <0.001 |
| BMI, kg/m^2^ | 22.85 (2.32) | 23.07 (2.51) | 0.309 |
| Systolic, mmHg | 110 (11) | 111 (11) | 0.541 |
| Diastolic, mmHg | 73 (8) | 73 (8) | 0.537 |
| Hypertension^a^ | 85 (47%) | 166 (47%) | 0.999 |
| Stage 1 | 74 (41%) | 153 (44%) | 0.652 |
| Stage 2 | 11 (6%) | 13 (4%) | 0.297 |
| Local home of residence (vs. migrant) | 62 (34%) | 135 (38%) | 0.366 |
| Well water source | 53 (29%) | 112 (32%) | 0.563 |
| Consumes alcohol | 9 (5%) | 16 (5%) | 0.772 |
| Smoker | 12 (7%) | 24 (7%) | 0.999 |
| Weeks worked first year | 26 (2) | 16 (10) | <0.001 |

^a^Stage 1 hypertension defined as systolic blood pressure ≥130 or diastolic blood pressure ≥ 80; Stage 2 hypertension defined as systolic blood pressure ≥140 or diastolic blood pressure ≥ 90
