# Supplementary material for: Longitudinal trends in renal function among first time sugarcane harvesters in Guatemala
Source: PLoS One. 2020 Mar 6;15(3):e0229413. doi: 10.1371/journal.pone.0229413 (PMC7059928; doi:10.1371/journal.pone.0229413)
Supplement: S2 Table — Presented as Odds Ratio (95% Confidence Interval). (DOCX) [file pone.0229413.s006.docx]

|  | Age-adjusted OR for Non-stable | p-value | Age- and baseline eGFR adjusted  OR for Non-stable | p-value |
| --- | --- | --- | --- | --- |
| Baseline Hypertension (N=85; 47%) | 2.80 (1.20, 6.74) | 0.020 | 5.21 (2.14, 13.94) | 0.001 |
| 20% Hypertension re-assigned (N=68; 38%) | 4.58 (1.71, 14.10) | 0.005 | 6.00 (2.24, 18.98) | 0.001 |
| 40% Hypertension re-assigned (N=51; 28%) | 0.60 (0.24, 1.54) | 0.278 | 0.54 (0.22, 1.33) | 0.171 |
