# Supplementary material for: Longitudinal trends in renal function among first time sugarcane harvesters in Guatemala
Source: PLoS One. 2020 Mar 6;15(3):e0229413. doi: 10.1371/journal.pone.0229413 (PMC7059928; doi:10.1371/journal.pone.0229413)
Supplement: Attachment — Submitted filename: Response to Reviewers.docx [file pone.0229413.s007.docx]

Dear Dr. Bolignano,

We would like to thank Dr. Provenzano for taking the time to provide feedback on our manuscript, *Longitudinal trends in renal function among first time sugarcane harvesters in Guatemala.* Below, in bold, we have addressed each point brought up by Dr. Provenzano as well as editorial concerns.

Reviewer #1: In Mesoamerica, chronic kidney disease of un-known origin (CKDu) is an important public health problem and this is because of several reasons: 1) This pidemic entity affects young and middle-aged men, resulting in high social-economic costs for health system. 2) Many causal risk factors have been hypothesized, but pathogenesis is still unexplained, therefore further studies are necessary. 3) Patients are often not aware of the disease, until CKDu reach final stages. Moreover, most patients have not the chance for renal replacement therapy and CKDu has become a leading cause of mortality. In this article Authors discuss on kidney disease development, trying to find new causes. One of the underlying factors is the presence of mild hypertension among this cohort, the unexpected prevalence of this latter, even among young men, raises new questions.

Comments:

- According to the esponential relationship between eGFR and creatinine, normal values of this latter can be related to an eGFR range between 50 and 180 ml/min. In other words, initial changes in eGFR determine little variations of creatinine, while pathological filtrates are associated to greater alterations of creatinine. Thus, resulting in a low sensitivity for this relationship in the earlier states of renal failure. The high current cut-off adopted by Company (serum creatinine level of 1.45 mg/dL or less) determines enrollment of individuals with impaired renal function. The Authors may concern about the need to decrease this value.

**Response: We agree that use of a creatinine of 1.45 mg/dL as a cutoff for hiring is too lax. In 2017, in response to our recommendations, the company changed the hiring cutoff to an eGFR above 90 ml/min per 1.73 m^2^. Despite the lenient hiring value during the time of our cohort construction, no worker in our analysis had a serum creatinine level above 1.20 mg/dL. The lowest observed baseline eGFR in our study was 84 ml/min per 1.73 m^2^. There were 5 individuals with a baseline eGFR below 90 ml/min per 1.73 m^2^ included in our analysis. We have updated the results section to mention this. We have also updated the conclusions to emphasize the need for a strict hiring cut-off.**

- The baseline covariates, defined at the screening visit, included: eGFR, age, body mass index (BMI), blood pressure, location of home residence, home drinking water source, alcohol consumption, and smoking status. However, potentially implicated factors have not been included, such as: daily water intake amount (dehydration could be contributing to heat stress), use of NSAIDs (very common among this populations). These latter should be mentioned in Limitations.

**Response: We agree that a limitation of our study was our inability to assess factors previously implicated in the development of CKDu. We have updated our limitations section to more clearly reflect this.**

- Regarding paragraph on pre-employment blood pressure measurements, it seems to be much descriptive. They should be summarized in some extents.

**Response: Thank you, we agree that we erred on the side of providing too much information and have summarized the blood pressure collection methodology.**

- The Authors found that mild hypertension (≥130/80 mmHg) could be one of the underlying causes of CKDu. This evidence is remarkable because other Authors had already depicted the linear relationship between blood pressure and kidney damage, even starting from normal values. However, the authors should mention that in non-adjusted and in age- and baseline eGFR- adjusted OR for non-stable eGFR, the study does not reach statistical significance on highly suspect causes implicated in CKDu such as: well water source, current or past smoking, alcohol consumption.

**Response**: **Thank you for pointing out this concern to us. We have updated the limitation section to mention our inability to test smoking and alcohol consumption. We have updated the results and discussion to mention an association with well water source.**

- Patients loss at the follow up can cause bias, workers may have been absent due to the kidney disease. It should be mentioned that loss can lead to underestimation of renal function decline, influencing relationship between eGFR and risk factor.

**Response: We agree that loss to follow-up and the “healthy worker effect” is a concern for research examining kidney function decline in worker cohorts. We have updated our limitations section to emphasize this.**

 Journal Requirements:

1. When submitting your revision, we need you to address these additional requirements.
Please ensure that your manuscript meets PLOS ONE's style requirements, including those for file naming. The PLOS ONE style templates can be found at
<http://www.journals.plos.org/plosone/s/file?id=wjVg/PLOSOne_formatting_sample_main_body.pdf> and <http://www.journals.plos.org/plosone/s/file?id=ba62/PLOSOne_formatting_sample_title_authors_affiliations.pdf>

**Response: We have updated the manuscript and supporting documents to meet the style requirements and file naming.**

2. Please include additional information regarding the survey or questionnaire used in the study and ensure that you have provided sufficient details that others could replicate the analyses. For instance, if you developed a questionnaire as part of this study and it is not under a copyright more restrictive than CC-BY, please include a copy, in both the original language and English, as Supporting Information.

**Response: We have included both a Spanish and English copy of the pre-employment survey that was developed and administered by the business in the supporting information. Full specifications on how we implemented the joint latent class models is provided in the supporting information.**

3. Thank you for stating the following in the Financial Disclosure section:

This evaluation was supported in part by Pantaleon; the Chancellor, CU Anschutz; Centers for Disease Control and Prevention (CDC.gov) (U19 OH01127 to LSN) and National Institutes of Health (NIH.gov) (R21 ES028826 to LSN). Its contents are solely the responsibility of the authors and do not necessarily represent the official views of the Centers for Disease Control and Prevention, the National Institutes of Health, or the Department of Health and Human Services.
The funders had no role in the evaluation design, data analysis, or data interpretation. Co-authors employed by the company (AC, CA, WDP) participated on the writing team. They provided details regarding work practices, but did not modify the results or conclusions of this report. The corresponding author had full access to all the raw data and had final responsibility for the decision to submit for publication. University of Colorado and Pantaleon are separate, independent organizations. University of Colorado employed appropriate research methods in keeping with academic freedom, based conclusions on critical analysis of the evidence and reported findings fully and objectively. The terms of this arrangement have been reviewed and approved by the University of Colorado in accordance with its conflict of interest policies.

We note that you received funding from a commercial source: Pantaleon

Please provide an amended Competing Interests Statement that explicitly states this commercial funder, along with any other [relevant declarations](http://www.plosone.org/static/competing.action) relating to employment, consultancy, patents, products in development, marketed products, etc.

Within this Competing Interests Statement, please confirm that this does not alter your adherence to all PLOS ONE policies on sharing data and materials by including the following statement: "This does not alter our adherence to PLOS ONE policies on sharing data and materials.” (as detailed online in our guide for authors <http://journals.plos.org/plosone/s/competing-interests>).  If there are restrictions on sharing of data and/or materials, please state these. Please note that we cannot proceed with consideration of your article until this information has been declared.

Please include your amended Competing Interests Statement within your cover letter. We will change the online submission form on your behalf.

Please know it is PLOS ONE policy for corresponding authors to declare, on behalf of all authors, all potential competing interests for the purposes of transparency. PLOS defines a competing interest as anything that interferes with, or could reasonably be perceived as interfering with, the full and objective presentation, peer review, editorial decision-making, or publication of research or non-research articles submitted to one of the journals. Competing interests can be financial or non-financial, professional, or personal. Competing interests can arise in relationship to an organization or another person. Please follow this link to our website for more details on competing interests: <http://journals.plos.org/plosone/s/competing-interests>

**Response: We have updated the Competing Interests Statement to reflect that our affiliation with Pantaleon does not alter our adherence to PLOS ONE policies on sharing data and materials and have included this in our cover letter.**

4. We note that you have stated that you will provide repository information for your data at acceptance. Should your manuscript be accepted for publication, we will hold it until you provide the relevant accession numbers or DOIs necessary to access your data. If you wish to make changes to your Data Availability statement, please describe these changes in your cover letter and we will update your Data Availability statement to reflect the information you provide.

**Response: We have no changes to address currently.**
